# Genome-Wide Association Studies for Dynamic Plant Height and Number of Nodes on the Main Stem in Summer Sowing Soybeans

**DOI:** 10.3389/fpls.2018.01184

**Published:** 2018-08-20

**Authors:** Fangguo Chang, Chengyu Guo, Fengluan Sun, Jishun Zhang, Zili Wang, Jiejie Kong, Qingyuan He, Ripa A. Sharmin, Tuanjie Zhao

**Affiliations:** National Center for Soybean Improvement, Key Laboratory of Biology and Genetics and Breeding for Soybean, Ministry of Agriculture, State Key Laboratory for Crop Genetics and Germplasm Enhancement, Nanjing Agricultural University, Nanjing, China

**Keywords:** soybean, genome-wide association study, quantitative trait nucleotide, plant height, number of nodes on the main stem, dynamic development

## Abstract

Plant height (PH) and the number of nodes on the main stem (NN) serve as major plant architecture traits affecting soybean seed yield. Although many quantitative trait loci for the two traits have been reported, their genetic controls at different developmental stages in soybeans remain unclear. Here, 368 soybean breeding lines were genotyped using 62,423 single nucleotide polymorphism (SNP) markers and phenotyped for the two traits at three different developmental stages over two locations in order to identify their quantitative trait nucleotides (QTNs) using compressed mixed linear model (CMLM) and multi-locus random-SNP-effect mixed linear model (mrMLM) approaches. As a result, 11 and 13 QTNs were found by CMLM to be associated with PH and NN, respectively. Among these QTNs, 8, 3, and 4 for PH and 6, 6, and 8 for NN were found at the three stages, and 3 and 6 were repeatedly detected for PH and NN. In addition, 34 and 30 QTNs were found by mrMLM to be associated with PH and NN, respectively. Among these QTNs, 11, 13, and 16 for PH and 11, 15, and 8 for NN were found at the three stages. A majority of these QTNs overlapped with the previously reported loci. Moreover, one QTN within the known *E2* locus for flowering time was detected for the two traits at all three stages, and another that overlapped with the *Dt1* locus for stem growth habit was also identified for the two traits at the mature stage. This may explain the highly significant correlation between the two traits. Our findings provide evidence for mixed major plus polygenes inheritance for dynamic traits and an extended understanding of their genetic architecture for molecular dissection and breeding utilization in soybeans.

## Introduction

Soybean (*Glycine max* L. Merr.) is an important source of plant protein and oil for human consumption. Improving seed yield is the major target for soybean breeders. Plant architecture can strongly affect the suitability and productivity of seed yield in agricultural crops (Li R. et al., 2014). Plant height (PH) and the number of nodes on the main stem (NN) as key plant type traits have obvious effects on the seed yield of soybean because they are related to some important characteristics such as lodging and adaptability (Chapman et al., 2003; Liu et al., 2011). PH and NN are highly correlated with some other soybean agronomic traits such as days to flowering (DF) and days to maturity (DM), which are thought to be mainly adaptive traits in response to the photoperiod, allowing each cultivar to adapt to limited geographic regions (Zhang et al., 2004, 2015; Panthee et al., 2007).

Plant height and NN are complex traits governed by many quantitative trait loci (QTL) in soybeans (Lee et al., 1996; Zhang et al., 2004; Liu et al., 2011, 2013; Yao et al., 2015; Cao et al., 2017). To date, more than 200 and 30 QTLs for PH and NN have been reported on SoyBase^1^ via linkage mapping. Recently, a study showed that highly significant correlations were observed among yield-related traits such as PH and NN. In addition, 23 novel QTLs and 8 QTL hotspots were identified for yield and quality-related traits by QTL analysis in a soybean RILs population. In particular, most loci associated with these traits were co-located in the same genomic region on three chromosomes (Chr04, Chr06, and Chr19), which was consistent with the results of phenotypic correlation analysis (Liu et al., 2017). Fang et al. (2017) identified 245 loci for 84 agronomic traits via genome-wide association studies (GWAS) in 809 soybean accessions and further dissected the genetic networks underlying the phenotypic correlations of traits. Of these traits, PH and NN exhibited a significant positive correlation. Some major genes were also cloned to reveal the molecular mechanism of PH and NN. Two known loci, *Dt1* for stem growth habit and *E2* for DF, were involved in regulating PH and NN and other agronomic traits in soybeans (Kato et al., 2015; Zhang et al., 2015). *Dt1* plays a primary role in determinate stem varieties and has an epistasis effect on the *Dt2* locus, another stem growth habit locus involved in the development of PH in soybeans (Bernard, 1972; Liu et al., 2016). The *E*2 locus encodes a homolog of *GIGANTEA*, which regulates the expression of *CO* and *FT* in *Arabidopsis* and controls soybean flowering through regulating *GmFT2a* (Watanabe et al., 2011). On the other hand, a target trait such as PH or NN performs dynamically when plants grow gradually. However, the phenotypes of PH and NN were mostly investigated at the mature stage. Sun et al. (2006) reported that different QTL architectures have been found for PH at the different developmental stages through linkage mapping. Although several studies of the developmental behavior of quantitative traits have been reported in soybeans (Vodkin et al., 2004; Li W. et al., 2007; Xin et al., 2008; Teng et al., 2009), the genetic architecture of dynamic development behavior of complex traits remains to be further explored.

With the wide application of next-generation sequencing techniques, high-throughput single nucleotide polymorphism (SNPs) have been discovered and utilized to construct high-resolution genetic maps and to conduct GWAS (Hyten et al., 2008; Michael and VanBuren, 2015; Song et al., 2016). GWAS is a powerful approach because it takes full advantage of all recombination events that occur in the evolutionary process of a natural population. It has been successfully used to explore the genetic basis for a broad range of complex traits in many plant species such as *Arabidopsis* (Atwell et al., 2010; Horton et al., 2012), rice (Huang et al., 2010; Yang et al., 2014), maize (Kump et al., 2011; Li H. et al., 2013), and soybean (Hwang et al., 2014; Sonah et al., 2015; Zhou et al., 2015; Zhang et al., 2016; Chang and Hartman, 2017).

The mixed linear models (MLMs) have been widely used for GWAS. The compressed MLM (CMLM) was also utilized to reduce computing time and to improve statistical power for quantitative trait nucleotide (QTN) detection (Zhang et al., 2010). Nevertheless, the current GWAS methods such as MLM and CMLM are mainly based on the single-locus genome-wide scan, which often requires correction for multiple tests. The typical Bonferroni correction is so conservative that some small-effect loci may not reach the significance threshold. With the rapid development of statistical methods, several multi-locus GWAS approaches have been developed to improve the power of QTN detection (Cho et al., 2010; Segura et al., 2012; Moser et al., 2015). The obvious advantage of these methods is no Bonferroni correction due to the nature of multi-locus methods. Recently, Wang et al. (2016) proposed a new multi-locus random-SNP-effect mixed linear model (mrMLM) method to improve the power and accuracy of GWAS. Differing from the other multi-locus methods, the mrMLM is a two-stage method. At the first stage, the SNP effect is viewed as being random, and all the potentially associated markers are selected by a random-SNP-effect MLM with a modified Bonferroni correction for significance test. At the second stage, all the selected markers are placed into one model and all the non-zero effects are further detected by a likelihood ratio test for QTN identification.

Summer-planting soybean is a major soybean crop grown in the region between the Yangtze River and the Huai River in the southern region of middle China, an important soybean production area. Although the genetic architecture of some agronomic traits such as PH was reported in our previous study (Cao et al., 2017) in the summer planting soybean, the genetic bases of dynamic PH and NN for them remain largely unknown. The aim of this study was to dissect the genetic basis of PH and NN at three different developmental stages in 368 summer planting soybean genotypes using the GWAS strategy. Our findings will provide useful genetic information for soybean molecular breeding.

## Materials and Methods

### Plant Materials, Field Trials and Phenotypic Evaluation

A soybean breeding line (SBL) population containing 368 accessions was established to service the local soybean breeding. All these pure lines were obtained from the National Center for soybean improvement, Nanjing Agricultural University, Nanjing, China. All experimental materials were planted at Jiangpu (JP) (32°12′N and 118°37′E) and Fengyang (FY) (32°47′N and 117°19′E) Station in Jiangsu and Anhui province, respectively, on June 20, 2011. At each location, the experimental design was a randomized complete block with 50 cm × 50 cm hill plots and three replications. The phenotypes for PH and NN were measured at the three different developmental stages over two locations: Stage 1 (35 days after the emergence of seedlings), Stage 2 (50 days after the emergence of seedlings) and Stage 3 (90 days after the emergence of seedlings). All the phenotypes were named PH1, PH2 and PH3 for PH, and NN1, NN2 and NN3 for NN. PH and NN were the averages of three measurements per plot.

### Statistical Analysis

The analysis of variance (ANOVA) was performed for all traits using the PROC GLM procedure of SAS version 9.3 (SAS Institute, Inc., Cary, NC, United States). The model for the phenotype of a trait was $y_{ijk}=\mu +G_{i}+E_{j}+GE_{ij}+R_{k\left(j\right)}+e_{ijk}$ where μ is the total mean, *G*_i_ is the effect of the *i*^th^ genotype, *E*_j_ is the effect of the *j*^th^ environment, GE_ij_ is the interaction effect between the *i*^th^ genotype and the *j*^th^ environment, R_k(j)_ is the effect of the *k*^th^ block within the *j*^th^ environment, and e_ijk_ is a random error following $N\left(0,\;\sigma_{e}^{2}\right)$. Descriptions of all traits were determined by the mean of each trait over two locations. The broad-sense heritability (*h*^2^) was calculated as: $h^{2}=\sigma_{g}^{2}/(\sigma_{g}^{2}+\sigma_{ge}^{2}/n+\sigma_{e}^{2}/nr)$ for combined environments and $h^{2}=\sigma_{g}^{2}/(\sigma_{g}^{2}+\sigma_{ge}^{2}/n+\sigma_{e}^{2}/nr)$ for an individual environment, where $\sigma_{g}^{2}$ is the genotypic variance, $\sigma_{ge}^{2}$ is the genotype by environment interaction variance, $\sigma_{e}^{2}$ is the error variance, *n* is the number of environments, and *r* is the number of replications. Variance components and correlation coefficients were estimated by the PROC VARCOMP and CORR procedure of SAS, respectively. To minimize the effects of environmental variation, the best linear unbiased predictors (BLUPs) of individual lines for each trait were calculated using the R package lme4 (Bates et al., 2015).

### Genotyping, SNPs Polymorphism and Haplotype Block Estimation

High-throughput SNPs were generated by RAD-seq. The quality control of sequencing data and methods of calling variations are described in our previous study (Li et al., 2016). A total of 62423 SNPs with a minor allele frequency (MAF) ≥ 5% were used for further analysis in the present study.

The MAF of the SNPs was calculated using VCFtools software (Danecek et al., 2011). Haplotype blocks were estimated using pLINK V1.90 software (Purcell et al., 2007) with the command option –blocks, following the default algorithm as described by Gabriel et al. (2002). The visualization of haplotype blocks was carried out with the R package LDheatmap (Shin et al., 2006). The estimated parameters for SNPs polymorphism were displayed using circos (Krzywinski et al., 2009).

### Linkage Disequilibrium Estimation

Linkage disequilibrium (LD) between pairwise SNPs was calculated as the squared correlation coefficient (*r*^2^) of alleles using the linkage disequilibrium tools option of RTM-GWAS V1.1 software (He et al., 2017). The *r*^2^ value was calculated for all pairwise SNPs with a 100 kb summary bin setting within the 5 Mb distance and then averaged across the whole genome. Because of the substantial difference in recombination rate between euchromatic and heterochromatic regions, the *r*^2^ value was calculated separately for the two chromosomal regions. The physical length of the euchromatic and heterochromatic regions for each chromosome was defined as in the *G. max* 1.0 reference genome. The LD decay rate was measured as the chromosomal distance at which the average pairwise *r*^2^ dropped to half its maximum value (Huang et al., 2010). Only *r*^2^ for SNPs with pairwise distances less than 5 Mb in either the euchromatic or heterochromatic region was used to draw the average LD decay figure by R script.

### Population Structure and Principal Component Analysis

Filtering SNPs used the –indep-pairwise command option of pLINK. The pruned data were then used to estimate population structure using ADMIXTURE V1.3.0 software (Alexander et al., 2009). In the ADMIXTURE setting, the number of clusters (*K*) was set from 1 to 10 initially; then, each Q and the relevant *P*-value was estimated. The most likely number of subpopulations was determined using the method described in Evanno et al. (2005). A principal component analysis (PCA) of whole-genome SNPs was performed using EIGENSOFT V5.0.2 software (Price et al., 2006) smartpca program, and the first two eigenvectors were plotted in two dimensions. The neighbor-joining tree was constructed using TASSEL V5.2 software (Bradbury et al., 2007).

### Genome-Wide Association Studies

After excluding SNPs with an MAF < 5%, 62423 SNPs were retained for 368 soybean accessions. To minimize false positives and increase statistical power, the population structure (Q) and kinship (K) matrix were estimated for the population. For the MLM, both the regular MLM and compressed CMLM involve the Q and K matrices as a fixed effect and random effect, where the Q matrix was replaced by the principal components (PCs) in CMLM. CMLM was implemented by the R package GAPIT (Genome Association Prediction Tool) V2 (Tang et al., 2016). Another R package, mrMLM V2.1, representing the mrMLM method was adopted (Wang et al., 2016). Thus, GWAS was conducted by combining the CMLM and mrMLM methods in this study. The critical threshold of significance for SNP-trait association was set at a *P*-value = 1.0 × 10^-4^ in CMLM according to the empirical value and at a LOD value of 3 in mrMLM. A QTN was defined as a haplotype block possessing SNPs identified as significantly associated with a trait (Schneider et al., 2016). The QTNs were named following the nomenclature described by McCouch et al. (1997). In addition, the abbreviation was used for the loci associated with the traits at the different stages. Thus, *qPH(NN)(1,2,3)-10-1* indicated a locus located on chromosome 10 associated with both PH and NN at all three stages.

### Prediction of Candidate Genes

Genes annotated in *G. max* Williams 82 reference gene model 1.0 were used as the source of candidate genes. The prediction of candidate genes mainly referred to the genes with a known function in soybeans related to the trait or the orthologs in *Arabidopsis*.

## Results

### Trait Performance of the Tested Population

The phenotypic characteristics of PH and NN for the 368 soybean lines are shown in **Table 1**. Averaged over two environments, PH and NN showed a large variation at the three different stages with range values of 27.97–109.06 (cm) and 7.67-20.39, respectively. The absolute values of kurtosis and skewness were approximately 1 for both PH and NN (**Supplementary Figure S1**). Significant positive correlations were observed for PH and NN among the three stages and between PH and NN (**Supplementary Figure S2**) (*r* > 0.60, *P* < 0.0001). PH1 and PH3 were moderately correlated with NN3 and NN1 at *r* = 0.49 and 0.48, *P* < 0.0001, respectively. Analysis of variance (ANOVA) indicated that there were significant differences in the effects of genotypes, environments, and their interactions for the traits at all stages. Additionally, a relatively high heritability (≥60%) was estimated for PH and NN at all stages, indicating that the genetic effects play a primary role in the performance of PH and NN.

**Table 1:** Descriptive statistics for plant height (cm) and number of nodes on the main stem at three stages over two environments in the SBL population.

Trait	Stage	Mean	*SD*	Minimum	Maximum	Skewness	Kurtosis	CV (%)	*F*_G_	*F*_E_	*F*_R(E)_	*F*_G__×__E_	*h*^2^ (%)
PH	1	50.48	9.95	27.97	90.22	0.80	1.06	19.70	9.99^∗∗∗^	84.43^∗∗∗^	24.33^∗∗∗^	1.53^∗∗∗^	84.97
	2	65.96	10.41	39.53	109.00	0.53	1.17	15.78	6.90^∗∗∗^	271.97^∗∗∗^	35.33^∗∗∗^	1.63^∗∗∗^	76.57
	3	74.39	12.45	41.28	109.06	0.13	-0.12	16.74	8.14^∗∗∗^	664.00^∗∗∗^	36.17^∗∗∗^	1.85^∗∗∗^	77.70
NN	1	10.21	1.32	7.67	15.94	0.84	0.74	12.88	7.39^∗∗∗^	52.21^∗∗∗^	11.19^∗∗∗^	1.28^∗∗^	83.10
	2	12.86	1.29	8.97	16.89	0.21	0.25	10.06	5.59^∗∗∗^	717.53^∗∗∗^	19.38^∗∗∗^	2.27^∗∗∗^	60.00
	3	14.58	1.59	9.58	20.39	0.16	0.74	10.91	5.41^∗∗∗^	2323.61^∗∗∗^	17.10^∗∗∗^	1.76^∗∗∗^	68.07


*F_*G*_, F_*E*_, F_*R(E)*_ and F_*G*__×__*E*_ represent the F-values for genotypic, environmental, block effects and genotype × environment interaction, respectively. Broad-sense heritability: $h^{2}=\sigma_{g}^{2}/(\sigma_{g}^{2}+\sigma_{ge}^{2}/n+\sigma_{e}^{2}/nr)$, where σ*_ge_^2^* is the genotypic variance, $\sigma_{ge}^{2}$ is the genotype by environment interaction variance, $\sigma_{e}^{2}$ is the error variance, n is the number of environments, r is the number of replications. ^∗∗^P < 0.001, ^∗∗∗^P < 0.0001.*

### Characterization of the SNPs, Population Structure, LD, and LD Haplotype Block Estimation

**FIGURE 1:**
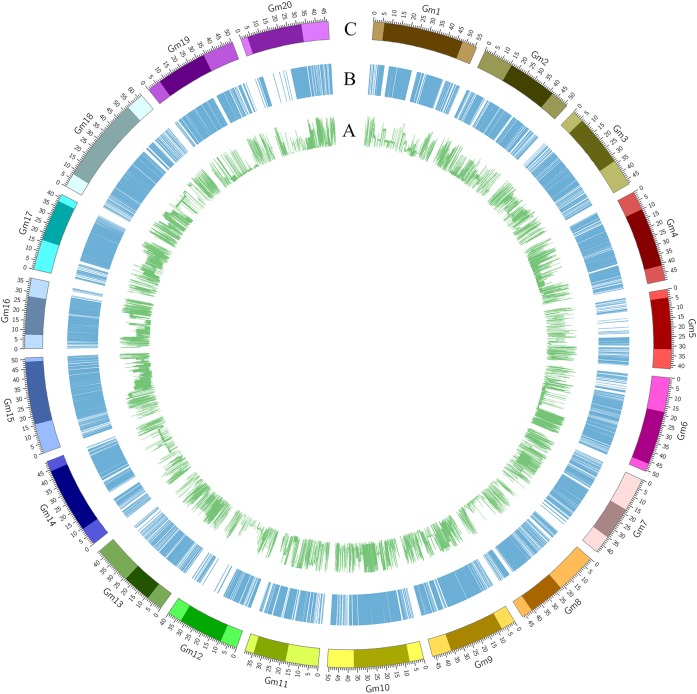
Characterization of the SNPs in the soybean genome. **(A)** Minor allele frequency of SNPs across the whole genome. **(B)** Distribution of LD blocks (>50 kb) in the whole genome. **(C)** Chromosomal region with pericentromeric regions in a darker color and whole chromosome in a lighter color (distance unit is Mb).

**FIGURE 2:**
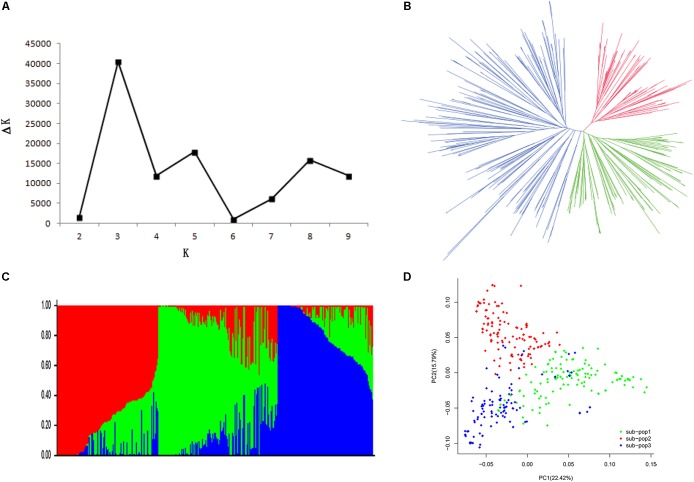
Population structure of 368 soybean accessions. **(A)** Calculation of the true *K* of the SBL population according to Evanno et al. (2005). **(B)** A neighbor-joining tree of the tested accessions that could be divided into three subpopulations. **(C)** Population structure was estimated by ADMIXTURE. Three colors represent three subpopulations, respectively. Each vertical column represents one individual and each colored segment in each column represents the percentage of the individual in the population. **(D)** PCA plot of the 368 accessions; two-dimensional scales were used to reveal population stratification.

**FIGURE 3:**
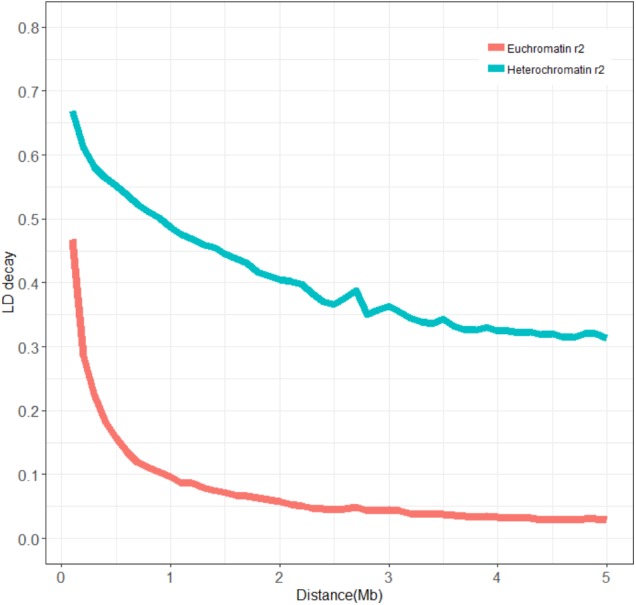
Average LD decay rates in euchromatic and heterochromatic regions of the whole genome. The mean LD decay rate was estimated as the squared correlation coefficient (*r*^2^) using all pairs of SNPs located within 5 Mb of physical distance in euchromatic (red) and heterochromatic (green) regions in the SBL population.

A total of 62423 SNPs with an MAF ≥ 0.05 were used for further analyses, with an average marker density of 1 SNP every 16.42 kb genome-wide, varying across chromosomes from 29.39 kb per SNP on chromosome 5 to 10.51 kb per SNP on chromosome 15. The MAF and haplotype block (>50 kb) for the population characteristics are presented in **Figure 1**. The most likely *K*-value was *K* = 3 based on the analysis of population structure (**Figure 2A**), which suggested that the overall population could be divided into three subpopulations. This result was also supported by the phylogenetic analysis (**Figures 2B,C**) and PCA (**Figure 2D**). The LD decay rate of the population was estimated at 400 kb in euchromatin, where *r*^2^ dropped to half of its maximum value (*r*^2^= 0.23) (**Figure 3**). In heterochromatin, however, *r*^2^ did not drop to half of its maximum value until 3.5 Mb. The haplotype analysis showed that 62423 SNPs were grouped into 5697 haplotype blocks. The size of the blocks ranged from 6 bp to 200 kb across the whole genome. The distribution of haplotype blocks is shown in **Supplementary Figure S3**.

### GWAS for the Traits via CMLM

**FIGURE 4:**
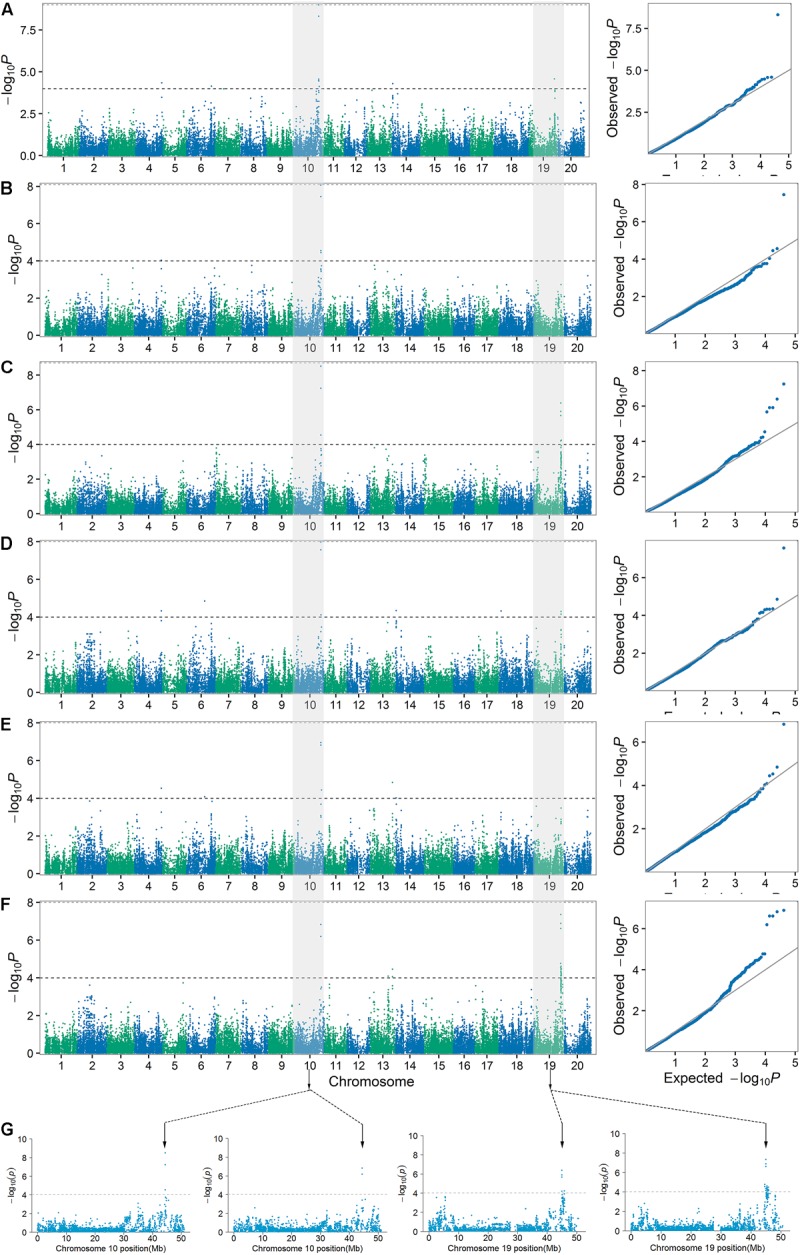
Manhattan plots and quantile–quantile plots for PH1 **(A)**, PH2 **(B)**, PH3 **(C)**, NN1 **(D)**, NN2 **(E)**, and NN3 **(F)** over three stages in the SBL population. The major loci for PH3 (left) and NN3 (right) in their Manhattan plots were located on chromosomes 10 and 19, respectively **(G)**.

Genome-wide association studies was conducted using the BLUPs of individual performance over two environments. A total of 11 loci for PH and 13 loci for NN were identified in the CMLM association panel at the suggestive significance level (*P* = 1 × 10^-4^). There were 8, 3, and 4 loci for PH and 6, 6, and 8 loci for NN at the three developmental stages. Three and six loci were detected for PH and NN at more than two stages, respectively (**Table 2** and **Figure 4**). Notably, two major loci associated with PH and NN were identified under the Bonferroni correction for multiple tests (0.05/*N*) (bold in **Table 2**). *qPH (NN)(1,2,3)-10-1* was identified on chromosome 10 at all stages, while *qPH(NN)3-19-1* was identified on chromosome 19 only at the last stage.

**Table 2:** Quantitative trait nucleotides (QTNs) associated with PH and NN via CMLM in the SBL population.

Trait	QTN	Chr	SNP	Position (bp)	Allele	MAF	-Log_10_ *P*^a^	*R^2^*^b^	*R^2^*^c^	Effect	Known QTL_S_^d^
PH	*qPH1-4-1*	4	Gm04_46680158	46680158	T/C	0.10	4.35	0.35	0.38	-3.71	5-4
	*qPH1-6-1*	6	Gm06_43710729	43710729	G/A	0.27	4.15	0.35	0.38	-3.08	3-2,6-3
	*qPH1-10-1*	10	Gm10_44226599	44226599	A/C	0.13	4.48	0.35	0.38	3.03	18-2,19-2
	***qPH1-10-2*^e^**	10	Gm10_44346474	44346474	A/T	0.41	9.26	0.35	0.42	3.57	*E2*
	*qPH1-10-3*	10	Gm10_44550928	44550928	C/T	0.33	4.45	0.35	0.38	-2.36	18-2,19-2
	*qPH1-10-4*	10	Gm10_45479097	45479097	C/T	0.11	4.13	0.35	0.38	3.31	
	*qPH1-14-1*	14	Gm14_926343	926343	G/A	0.21	4.29	0.35	0.38	2.40	34-6
	*qPH1-19-1*	19	Gm19_44544574	44544574	A/G	0.36	4.58	0.35	0.38	-2.53	1-1
	*qPH2-4-1*	4	Gm04_46680158	46680158	T/C	0.10	4.04	0.29	0.32	-3.36	5-4
	***qPH2-10-2***	10	Gm10_44346474	44346474	A/T	0.41	8.07	0.29	0.36	3.13	*E2*
	*qPH2-10-3*	10	Gm10_44550928	44550928	C/T	0.33	4.45	0.29	0.32	-2.24	18-2,19-2
	***qPH3-10-2***	10	Gm10_44346474	44346474	A/T	0.41	8.52	0.25	0.33	3.80	*E2*
	***qPH3-19-2***	19	Gm19_44938780	44938780	C/T	0.35	6.39	0.25	0.31	3.74	*Dt1*
	*qPH3-19-3*	19	Gm19_45721414	45721414	G/T	0.33	4.25	0.25	0.29	-2.62	3-1,4-2,6-1
	*qPH3-20-1*	20	Gm20_41211643	41211643	G/T	0.12	4.02	0.25	0.29	3.03	28-1
NN	*qNN1-4-1*	4	Gm04_46680158	46680158	T/C	0.10	4.33	0.34	0.37	-0.46	
	*qNN1-6-1*	6	Gm06_31222865	31222865	C/T	0.10	4.85	0.34	0.38	-0.52	4-2
	***qNN1-10-1***	10	Gm10_44346474	44346474	A/T	0.41	7.99	0.34	0.40	0.41	*E2*
	*qNN1-14-1*	14	Gm14_926343	926343	G/A	0.21	4.35	0.34	0.37	0.30	
	*qNN1-18-1*	18	Gm18_3716679	3716679	T/C	0.21	4.32	0.34	0.37	0.39	
	*qNN1-19-1*	19	Gm19_45415096	45415096	C/A	0.20	4.30	0.34	0.37	-0.34	
	*qNN2-4-1*	4	Gm04_46680158	46680158	T/C	0.10	4.53	0.29	0.33	-0.27	
	*qNN2-6-1*	6	Gm06_31222865	31222865	C/T	0.10	4.08	0.29	0.32	-0.28	4-2
	***qNN2-10-1***	10	Gm10_44346474	44346474	A/T	0.41	6.95	0.29	0.35	0.22	*E2*
	*qNN2-10-2*	10	Gm10_45479097	45479097	C/T	0.11	4.44	0.29	0.33	0.25	
	*qNN2-13-1*	13	Gm13_38510582	38510582	T/G	0.13	4.84	0.29	0.33	0.21	
	*qNN2-14-1*	14	Gm14_926343	926343	G/A	0.21	4.02	0.29	0.32	0.17	
	***qNN3-10-1***	10	Gm10_44346474	44346474	A/T	0.41	6.83	0.25	0.31	0.11	*E2*
	*qNN3-13-2*	13	Gm13_31053641	31053641	T/C	0.25	4.09	0.25	0.29	-0.09	1-8
	*qNN3-13-1*	13	Gm13_38510582	38510582	T/G	0.13	4.46	0.25	0.29	0.10	
	*qNN3-19-2*	19	Gm19_44558007	44558007	C/A	0.34	4.77	0.25	0.29	-0.09	
	***qNN3-19-3***	19	Gm19_44938780	44938780	C/T	0.35	7.36	0.25	0.32	0.13	*Dt1*
	*qNN3-19-4*	19	Gm19_45295148	45295148	C/G	0.39	4.61	0.25	0.29	0.09	
	*qNN3-19-1*	19	Gm19_45384848	45384848	A/G	0.40	4.10	0.25	0.29	-0.08	
	*qNN3-19-5*	19	Gm19_45727395	45727395	G/A	0.39	4.48	0.25	0.29	-0.09	


*^*a*^Negative log_*10*_-transformed P-value of the suggestive. ^*b*^The contribution rate of the model without SNP. ^*c*^The contribution rate of the model with SNP. ^*d*^Based on the QTL list on SoyBase (www.soybase.org). ^*e*^The QTNs shown in bold indicate that they reach the significance threshold based on the Bonferroni correction.*

**FIGURE 5:**
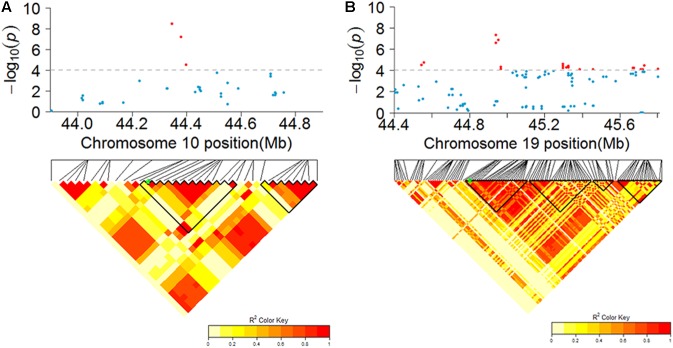
Candidate regions of the genome showing significant association signals near identified major loci for PH and NN. The top of each panel shows the Manhattan plot indicating the level of SNP association with PH **(A)** or NN **(B)**. Gray horizontal dashed lines indicate the genome-wide suggestive threshold. The bottom of each panel shows the local LD of the chromosomal regions containing the peak SNP (SNP with the lowest *P*-value), whose position is indicated by a green asterisk. Nearby haplotype blocks are outlined in black triangles.

The haplotype analysis showed that the peak SNPs for these two major loci were located within the two haplotype blocks named H2842 and H5441. Three main haplotypes (total frequency > 80%), H2842-1, H2842-2, and H2842-3, were identified for H2842, whose frequencies were 19.02, 58.42, and 10.87%, respectively, and two main haplotypes, H5441-1 and H5441-2, were identified for H5441, with frequencies of 46.47% and 33.70%, respectively. We further analyzed the effect of these haplotypes for PH and NN. The results showed that there were significant differences for both PH and NN among the main haplotypes of H2842 or H5441 (**Supplementary Figure S4**). The LD and haplotype blocks for these two major loci are presented in **Figure 5**.

### GWAS for Traits via mrMLM

To validate the reliability of the loci determined by the CMLM method and identify more loci associated with PH and NN, a multi-locus random effect MLM method was used to conduct GWAS.

**FIGURE 6:**
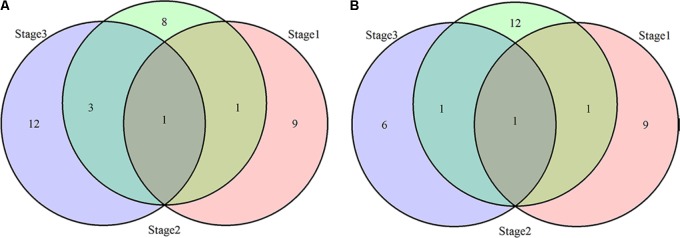
Venn diagrams for loci associated with PH **(A)** and NN **(B)** over three stages.

Thirty-four loci for PH and 30 loci for NN were identified using the MRMLM method. Among them, 11, 13, and 16 loci for PH and 11, 15, and 8 loci for NN were detected at the three developmental stages (**Tables 3, 4** and **Figure 6**). Due to the differential genetic control of PH and NN at the different stages, further comparative analysis showed that *qPH1(2)-10-1*, *qPH1(2)-13-5* and *qPH2(3)-7-1*, *qPH2(3)-9-3*, *qPH2(3)-10-1* and *qPH2(3)-13-1* were commonly detected for PH at the first and last two stages, respectively (bold in **Table 3**). *qNN1(2)-10-1*, *qNN1(2)-14-1* and *qNN2(3)-10-1*, *qNN2(3)-13-3* were commonly detected for NN at the first and last stages, respectively (bold in **Table 4**). It was clear that there were many differential loci for both PH and NN among the different stages. Similar to the CMLM results, *qPH (NN)(1,2,3)-10-1* as a major locus was identified and shared for PH and NN at three stages. The same peak SNP (Gm10_44346474, MAF = 0.41) could explain 13, 8, and 10% of the phenotypic variation for PH and 7, 4, and 11% of the phenotypic variation for NN at the three stages, respectively. *qPH (NN)3-19-1(3)*, another major locus, was detected for both PH and NN at the last stage. The same peak SNP (Gm19_44938780, MAF = 0.35) could explain 8 and 9% of the phenotypic variation for PH and NN at the last stage, respectively. In addition to these two major loci, *qPH1-4-1*, *qPH1-6-2*, and *qPH1-14-1* for PH and *qNN1-4-1*, *qNN2-13-3*, and *qNN1-14-1* for NN were validated by the MRMLM method. Moreover, four loci, *qPH1-4-1*, *qPH1-6-2*, *qPH2-13-1*, and *qPH1-14-1* for PH and *qNN1-4-1*, *qNN1-6-1*, *qNN3-13-2*, and *qNN1(2)-14-1* for NN, were co-located in the same genomic regions. Some novel loci were identified for PH and NN via the MRMLM method compared with the CMLM method and are shown in **Tables 3, 4**.

**Table 3:** QTNs associated with PH via mrMLM in the SBL population.

QTN	Chr	SNP	Allele	MAF	Position (bp)	Effect^a^	LOD score^b^	*R*^2^	Known QTL^c^
*qPH1-4-1*^d^	4	Gm04_46680158	T/C	0.10	46680158	-4.42	9.43	0.11	5-4
*qPH1-5-1*	5	Gm05_36588358	G/A	0.23	36588358	2.11	4.84	0.05	24-1
*qPH1-6-1*	6	Gm06_33125042	T/C	0.07	33125042	2.89	4.48	0.03	30-1
*qPH1-6-2*	6	Gm06_43710729	G/A	0.27	43710729	-2.52	5.64	0.07	3-2,6-3
*qPH1-6-3*	6	Gm06_48441344	A/G	0.19	48441344	1.76	6.01	0.03	38-1
*qPH1-9-1*	9	Gm09_38680990	C/T	0.45	38680990	1.20	3.73	0.02	
***qPH1-10-1*^e^**	10	Gm10_44346474	A/T	0.41	44346474	3.04	14.99	0.13	*E2*
***qPH1-13-5***	13	Gm13_41776586	C/T	0.05	41776586	3.04	4.11	0.03	39-1
*qPH1-14-1*	14	Gm14_1243675	A/G	0.21	1243675	1.92	4.59	0.04	34-6
*qPH1-18-1*	18	Gm18_7790416	T/C	0.34	7790416	-0.91	3.61	0.01	23-6
*qPH1-18-2*	18	Gm18_51243803	A/G	0.22	51243803	-1.66	3.26	0.03	26-12
*qPH2-6-4*	6	Gm06_13482496	G/A	0.36	13482496	-1.37	3.40	0.03	
***qPH2-7-1***	7	Gm07_37502305	T/C	0.35	37502305	1.59	4.15	0.04	37-6
*qPH2-8-1*	8	Gm08_41977134	G/A	0.42	41977134	1.30	3.01	0.03	
*qPH2-9-2*	9	Gm09_7987471	C/T	0.23	7987471	1.60	5.16	0.03	mqPH-009
***qPH2-9-3***	9	Gm09_41284917	G/A	0.05	41284917	2.42	3.55	0.02	
***qPH2-10-1***	10	Gm10_44346474	A/T	0.41	44346474	2.21	7.67	0.08	*E2*
***qPH2-13-1***	13	Gm13_6859748	A/G	0.46	6859748	1.37	4.05	0.03	20-5
*qPH2-13-2*	13	Gm13_8246449	T/C	0.48	8246449	2.04	8.31	0.07	20-5
*qPH2-13-3*	13	Gm13_10898897	T/C	0.50	10898897	-1.57	5.56	0.04	26-11
*qPH2-13-4*	13	Gm13_28457573	T/G	0.06	28457573	-6.23	9.92	0.13	5-8,15-1
***qPH2-13-5***	13	Gm13_41776586	C/T	0.05	41776586	3.49	5.58	0.04	39-1
*qPH2-14-2*	14	Gm14_3056277	T/C	0.19	3056277	2.04	6.30	0.04	34-6
*qPH2-20-1*	20	Gm20_41211643	G/T	0.12	41211643	2.12	4.68	0.03	28-1
*qPH3-2-1*	2	Gm02_2328263	C/A	0.20	2328263	-3.03	9.85	0.06	9-3
*qPH3-2-2*	2	Gm02_46786754	C/T	0.47	46786754	-1.67	3.98	0.03	6-12
*qPH3-3-1*	3	Gm03_1920671	A/G	0.26	1920671	1.70	4.19	0.02	
*qPH3-3-2*	3	Gm03_6757344	T/C	0.26	6757344	1.63	6.27	0.02	
*qPH3-6-5*	6	Gm06_31222865	C/T	0.10	31222865	2.25	4.44	0.02	30-1
*qPH3-7-2*	7	Gm07_984279	C/G	0.09	984279	2.56	3.46	0.02	
***qPH3-7-1***	7	Gm07_37502305	T/C	0.35	37502305	1.88	3.21	0.04	37-6
*qPH3-8-2*	8	Gm08_18166829	A/C	0.46	18166829	1.46	5.53	0.02	
***qPH3-9-3***	9	Gm09_41284917	G/A	0.05	41284917	2.93	3.96	0.02	
*qPH3-10-2*	10	Gm10_34971279	C/G	0.13	34971279	2.99	8.06	0.04	
***qPH3-10-1***	10	Gm10_44346474	A/T	0.41	44346474	3.02	15.21	0.10	*E2*
***qPH3-13-1***	13	Gm13_6859748	A/G	0.46	6859748	1.93	6.83	0.04	20-5
*qPH3-13-6*	13	Gm13_27311661	G/C	0.29	27311661	2.89	9.19	0.08	5-8,15-1
*qPH3-14-3*	14	Gm14_10617104	A/G	0.39	10617104	1.32	4.43	0.02	34-6
*qPH3-19-1*	19	Gm19_44938780	C/T	0.35	44938780	-2.81	14.27	0.08	*Dt1*
*qPH3-20-2*	20	Gm20_27547938	G/A	0.33	27547938	3.00	12.48	0.09	16-1


*^*a*^Quantitative trait nucleotide effect. ^*b*^LOD value, the significant threshold for the transformed P-value. ^*c*^The QTLs located in the same region of reported QTLs. ^*d*^The QTN identified for PH at the same stage by both methods are underlined in Table. ^*e*^The QTNs identified for PH at least two stages are displayed in bold in Table.*

**Table 4:** QTNs associated with NN via mrMLM in the SBL population.

QTN	Chr	SNP	Allele	MAF	Position (bp)	QTN effect	LOD score	*R*^2^	Known QTL
*qNN1-2-1*	2	Gm02_5017572	G/A	0.48	5017572	0.25	7.62	0.07	
*qNN1-4-1*^a^	4	Gm04_46680158	T/C	0.10	46680158	-0.37	7.84	0.05	
*qNN1-5-1*	5	Gm05_27229418	A/G	0.32	27229418	0.22	3.62	0.04	
*qNN1-6-1*	6	Gm06_43710729	G/A	0.27	43710729	-0.26	3.90	0.05	1-3,1-4,2-1
***qNN1-10-1*^b^**	10	Gm10_44346474	A/T	0.41	44346474	0.27	8.56	0.07	*E2*
*qNN1-10-2*	10	Gm10_45479097	C/T	0.11	45479097	-0.42	6.69	0.07	
***qNN1-14-1***	14	Gm14_1243675	A/G	0.21	1243675	0.20	4.48	0.03	
*qNN1-15-1*	15	Gm15_7957092	T/G	0.31	7957092	-0.25	5.99	0.06	
*qNN1-17-1*	17	Gm17_38445432	T/G	0.23	38445432	0.18	3.93	0.02	
*qNN1-17-2*	17	Gm17_38559614	T/C	0.07	38559614	-0.28	3.24	0.02	
*qNN1-19-1*	19	Gm19_2487047	C/T	0.11	2487047	-0.27	3.60	0.03	
*qNN2-4-2*	4	Gm04_9024569	T/C	0.39	9024569	0.16	8.26	0.07	5-1
*qNN2-6-2*	6	Gm06_14119587	C/T	0.06	14119587	0.18	3.28	0.02	7-2
*qNN2-6-3*	6	Gm06_17601986	C/A	0.18	17601986	-0.18	6.57	0.06	7-2
*qNN2-6-4*	6	Gm06_41843359	C/A	0.45	41843359	-0.09	3.02	0.02	1-3,1-4,2-1
*qNN2-8-1*	8	Gm08_18313651	T/A	0.17	18313651	0.13	4.01	0.03	
*qNN2-10-3*	10	Gm10_4827909	G/A	0.08	4827909	-0.16	4.01	0.03	
***qNN2-10-1***	10	Gm10_44378814	T/C	0.41	44378814	0.12	4.39	0.04	*E2*
*qNN2-12-1*	12	Gm12_8229718	C/T	0.23	8229718	0.11	3.08	0.02	
*qNN2-13-1*	13	Gm13_7126248	C/A	0.42	7126248	0.13	6.71	0.05	1-6
***qNN2-13-3***	13	Gm13_38510582	T/G	0.13	38510582	0.14	3.73	0.02	
***qNN2-14-1***	14	Gm14_1243675	A/G	0.21	1243675	0.12	3.99	0.03	
*qNN2-14-2*	14	Gm14_1557061	C/T	0.20	1557061	0.12	3.06	0.03	
*qNN2-18-1*	18	Gm18_8519411	C/T	0.34	8519411	0.16	6.78	0.07	
*qNN2-19-2*	19	Gm19_45415096	C/A	0.20	45415096	0.15	4.77	0.04	
*qNN2-20-1*	20	Gm20_40247542	G/A	0.32	40247542	0.09	3.24	0.02	
*qNN3-6-5*	6	Gm06_50072605	A/G	0.46	50072605	0.05	5.68	0.04	1-3
***qNN3-10-1***	10	Gm10_44346474	A/T	0.41	44346474	0.09	11.42	0.11	*E2*
*qNN3-11-1*	11	Gm11_8556480	G/A	0.22	8556480	-0.08	6.94	0.06	3-3,3-4,3-5,3-6
*qNN3-13-2*	13	Gm13_6859748	A/G	0.46	6859748	0.05	5.09	0.03	1-6
***qNN3-13-3***	13	Gm13_38510582	T/G	0.13	38510582	0.08	4.30	0.03	
*qNN3-14-3*	14	Gm14_11281151	T/C	0.40	11281151	0.08	7.22	0.08	
*qNN3-16-1*	16	Gm16_27302091	A/T	0.18	27302091	0.06	4.28	0.03	
*qNN3-19-3*	19	Gm19_44938780	C/T	0.35	44938780	-0.09	9.66	0.09	*Dt1*


*^*a*^The QTNs identified for NN at the same stage by both methods are underlined in Table. ^*b*^The QTNs identified for NN at least two stages are displayed in bold in Table.*

### Prediction of Candidate Genes

To predict candidate genes for loci significantly associated with both PH and NN, we selected the putative genes tagged by the most significant SNPs. Two major loci coassociated with PH and NN pinpointed the known genes. *E2*(*Glyma10g36600*), encoding a homolog of GIGANTEA (*GI*) protein, is one of the key genes regulating soybean flowering and maturity by regulating *GmFT2a* (Watanabe et al., 2011). It was found 370 kb upstream of the peak SNP (Gm10_44346474, MAF = 0.41) of *qPH1-10-1* (*qNN1-10-1*) on Gm10, which was associated with both PH and NN at all three stages (**Tables 3, 4**). *Dt1* (*Glyma19g37890*) is a homolog of *Arabidopsis TERMINAL FLOWER1* (*TFL1*) and plays a primary role in the soybean stem growth habit (Bernard, 1972). It was found 41 kb upstream of the peak SNP (Gm19_44938780, MAF = 0.35) of *qPH3-19-1* (*qNN3-19-3*) on Gm19, which was associated with both PH and NN at the last stage. In addition to these two major loci, we predicted the candidate genes for two other loci associated with both PH and NN. A putative gene, *Glyma04g40640*, was found 35 kb from the peak SNP (Gm04_46671367, MAF = 0.11) of *qPH1-4-1* (*qNN1-4-1*) on Gm04, which was associated with both PH and NN at the first stage. It was homologous to *Arabidopsis APRR5*, which is involved in various circadian-associated biological events such as flowering time in long-day photoperiod conditions and red light sensitivity of seedlings during early photomorphogenesis (Kamioka et al., 2016). The putative gene *Glyma13g06811* was identified 125 kb from the peak SNP (Gm13_6859748, MAF = 0.46) of *qPH2-13-1*(*qNN3-13-2*) on Gm13, which was associated with both PH and NN at the second and third stages, respectively. It is homologous to *Arabidopsis AGAMOUS-LIKE 8* (*AGL8*), which is a MADs-box transcription factor involved in various biological events such as flower and fruit development and the maintenance of inflorescence meristem characteristics (Ma et al., 1991).

## Discussion

### Linkage Disequilibrium and the Statistical Method Are of Great Significance in GWAS

Genome-wide association studies are a powerful tool to elucidate the genetic architecture for complex quantitative traits in crops (Morris et al., 2013; Su et al., 2016; Sun et al., 2017). The mapping resolution and statistical power are the main considerations in GWAS. LD is one of the factors limiting the mapping resolution of GWAS. The recombination rate is one of the major factors affecting LD extension and is different between euchromatic and heterochromatic regions (Zhang et al., 2015). Previous studies suggested that there was a very diverse range of LD values in different crops and different chromosomal regions in a specific crop (Li Y.H. et al., 2014; Sonah et al., 2015; Zhang et al., 2015). Similar to previous studies, a large difference in LD decay rate was also observed between the euchromatin (400 kb) and heterochromatin regions (3.5 Mb) of soybeans in this study. A longer LD has been observed in self-pollinated crops such as soybean compared to cross-pollinated crops such as maize (Li et al., 2016). A previous study also reported a longer LD for the chromosomes involved in the domestication process. The QTLs for domestication traits such as seed weight and flowering were mapped in these regions (Li Y.H. et al., 2013).

Another concern with GWAS is the statistical method. In the present study, the CMLM method was used for the single-locus GWAS. Consistent with the previous study, we also found that the CMLM method could take less computing time than regular MLM and reduce false positive results simultaneously (Zhang et al., 2015). However, the single-locus GWAS methods often need correction for multiple tests. For instance, the typical Bonferroni correction corrects an α = 0.05 to α = 0.05/*m*, where *m* is the number of statistical tests performed. For a GWAS with 500,000 markers, the statistical significance threshold for an association would be corrected to 1e^-7^, such that no or a few loci could reach the significance threshold after the correction. Such a situation is not always suited to the nature of complex traits. A previous study showed that no significantly associated locus for soybean seed weight was detected, possibly for this reason (Fang et al., 2017). Wang et al. (2016) also reported that some small-effect loci were not significantly associated with the traits in the single-locus approach under the Bonferroni correction but significantly associated with that in the mrMLM method. Actually, the small-effect loci should not be neglected in the genetic system of complex traits. Fortunately, several multi-locus GWAS methods have already been reported in previous studies (Segura et al., 2012; Wang et al., 2016), where no Bonferroni correction is needed because of the multi-locus nature. Thus, the multi-locus GWAS method may play an increasingly important role in dissecting the genetic architecture of complex traits in the post-association time.

### Combination of CMLM and mrMLM GWAS Methods to Identify the Major and Minor Loci for PH and NN in the SBL Population

Plant height and NN are quantitative traits controlled by numerous loci in soybeans^2^. In the present study, 11 loci for PH and 13 loci for NN were detected via the CMLM method at the suggestive threshold level, only two of which were identified after the Bonferroni correction. To confirm the loci determined by the CMLM method and identify additional loci for PH and NN, mrMLM as a multi-locus method was used for GWAS in this study. As expected, we found 32 and 28 loci, except for two major loci, for PH and NN via the mrMLM method. Some novel loci in comparison with that obtained from the CMLM were located around the previously reported QTLs. For PH, the loci *qPH1-18-1* and *qPH1-18-2* detected by the mrMLM method have been previously identified by Sun et al. (2006), which were also located in the same genomic regions of the known QTLs. However, only six loci in addition to the two major loci, *qPH1-4-1*, *qPH1-6-1*, and *qPH1-14-1* for PH and *qNN1-4-1*, *qNN1-14-1*, and *qNN2-13-1* for NN, identified at a suggestive threshold level (*P* = 10^-4^) in CMLM were validated in mrMLM association panel. One potential reason was that the mrMLM method improved the power and accuracy for QTN detection due to the nature of the statistical model. Thus, many novel loci were detected by the mrMLM method. Another possible reason was that the relatively stringent threshold still applied to the CMLM method. More loci might be commonly detected in two association panels if a lower threshold was adopted in the CMLM method, but the false positive results might increase under such conditions. Undoubtedly, mrMLM can identify not only the major loci but also the minor loci for quantitative traits compared with the CMLM method.

### Genetic System of Dynamic PH and NN in Summer Planting Soybeans

Many agronomic and yield-related traits such as PH and NN were highly correlated in soybeans. Previously identified loci for these traits usually co-located in the same genomic regions (Malik et al., 2007; Liu et al., 2011, 2017; Fang et al., 2017). Similarly, significant positive correlations were observed between PH and NN at different stages in this study. Furthermore, six loci, including major and minor loci, were shared for PH and NN, suggesting that both PH and NN have a similar genetic system controlled by major and minor loci. Previous studies showed that the *E2* and *Dt1* loci have an effect on many agronomic and yield-related traits in soybean (Liu et al., 2011; Kato et al., 2015; Zhang et al., 2015). We also found that PH and NN shared these two loci and further confirmed that the genetic pattern of the *E2* locus was different from that of the *Dt1* locus. The former was detected for both PH and NN at all stages while the latter was only detected at the last stage.

Plant height and NN in soybeans are dynamic traits, as the phenotype changes constantly during the plant lifecycle. However, studies of the genetic basis of PH and NN have mainly measured the final PH and number of nodes on the main stem, especially when the phenotype was investigated at the mature stage. We accessed PH and NN in the summer-planting accessions at three different stages, which included one vegetative stage and two reproductive stages when plants were in late vegetative growth, as well as the flowering and mature stages, to reveal the genetic control underlying the dynamic PH and NN based on the GWAS strategy in this study. A previous report showed that the haplotype was more appropriate than the single SNP to uncover the genetic variation and improve the efficiency of breeding for target traits due to the existence of multiple alleles (Hao et al., 2012). We identified the *E2* and *Dt1* genes in two haplotype blocks. The analysis of haplotypes revealed that the main haplotypes of these two haplotype blocks were related to PH and NN over stages. Our results suggested that *E2* and *Dt1*, as the major loci, play different roles in regulating the development of PH and NN at different stages. Nine and 20 novel loci were identified for PH and NN at different stages via a new multi-locus GWAS method, respectively. Moreover, the differential loci were identified for both PH and NN among the different stages. These common and specific loci for PH and NN at different stages unveil the genetic architecture underlying the dynamic PH and NN. Although most of them have not been confirmed yet, candidate genes were predicted for several loci associated with both PH and NN, some of which are located in the same regions as known QTLs for PH and NN in SoyBase. Further studies should confirm these loci and identify candidate genes for them.

## Conclusion

More loci, including 34 loci for PH and 30 loci for NN, were identified by the mrMLM method than by the single-locus CMLM method. A few loci were commonly identified for PH and NN via the two methods at the different developmental stages. One stable locus that overlapped with the *E2* gene was identified for PH and NN at all three stages, while another major locus, referred to as the *Dt1* gene, was determined at the last stage by both methods. Most loci were mainly detected at only one or two of the examined developmental stages. The dynamic PH and NN was controlled by a set of specific loci and a few common loci in summer planting soybeans.

## Author Contributions

TZ conceived and designed the experiments. CG, FS, JZ, JK, and QH performed the experiments. FC and ZW analyzed the data. FC and RS drafted the manuscript. TZ revised the paper.

## Conflict of Interest Statement

The authors declare that the research was conducted in the absence of any commercial or financial relationships that could be construed as a potential conflict of interest.

## Footnotes

1 www.soybase.org

2 www.soybase.org
