# Supplementary material for: Genome-Wide Association Studies for Dynamic Plant Height and Number of Nodes on the Main Stem in Summer Sowing Soybeans
Source: Front Plant Sci. 2018 Aug 20;9:1184. doi: 10.3389/fpls.2018.01184 (PMC6110304; doi:10.3389/fpls.2018.01184)
Supplement: Supplementary file 1 [file Table_1.docx]

**Supplementary materials**

**Supplementary Figure 1** Frequency distributions of the PH **(A)** and NN **(B)** traits in the SBL population. Shown is the average of each trait in the population over two environments across three stages each with three replicates.

**Supplementary Figure 2** Plot of the correlation coefficients of each pair of traits.

**Supplementary Figure 3** Distribution of the haplotype blocks among the whole genome.

**Supplementary Figure 4** The haplotype analysis of the two major loci for PH and NN at three stages.

**(A)** Comparison of the PH among the haplotypes of H2842 and the PH between the haplotypes of H5441 at three stages using a Duncan's multiple range test (*P<* 0.01) and an independent *t*-test (** *P*<0.01), respectively. **(B)** Comparison of the NN among the haplotypes of H2842 and the NN between the haplotypes of H5441 at three stages using a Duncan's multiple range test (*P<* 0.01) and an independent *t*-test, respectively.

**Supplementary Figure 1**

**
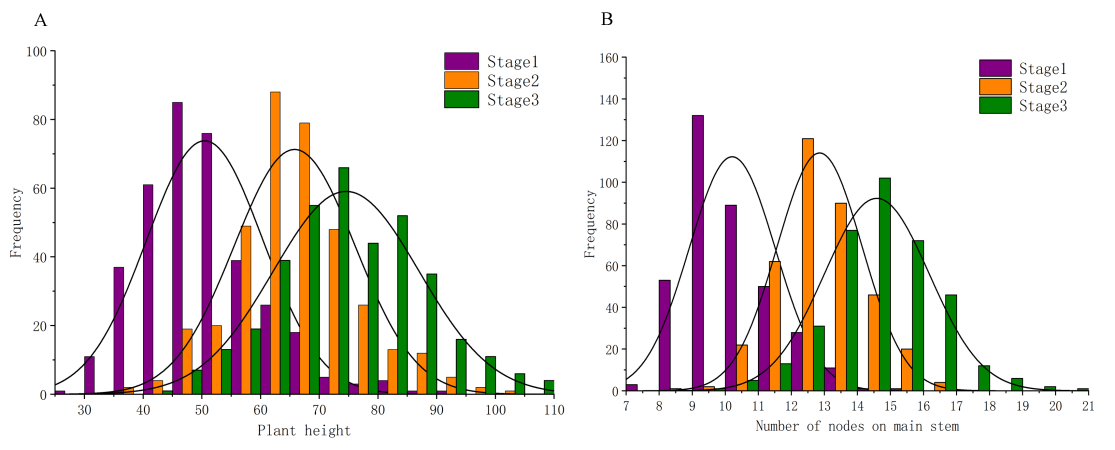
**

**Supplementary Figure 2**

**
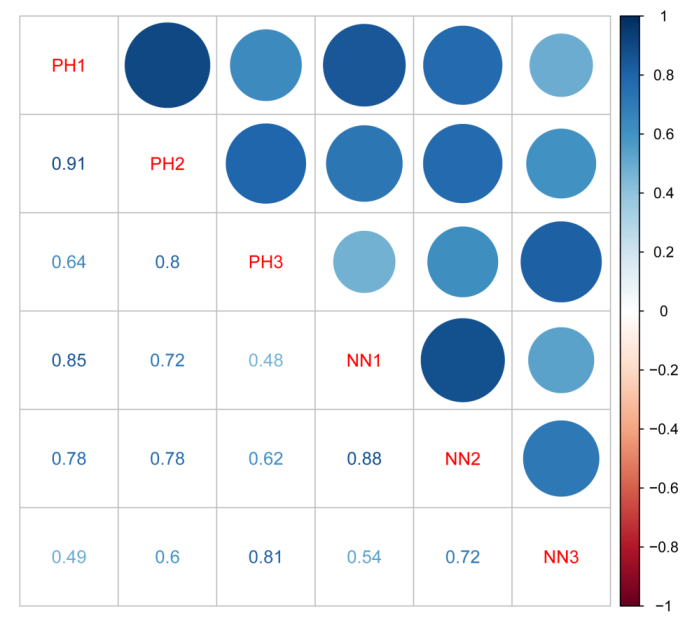
**

Significant at *P*<0.0001

**Supplementary Figure 3**

**
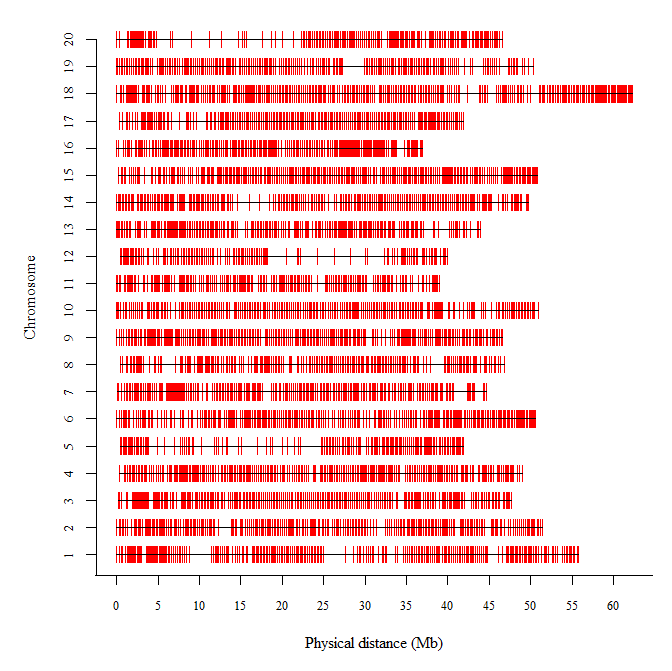
**

**Supplementary Figure 4**

**
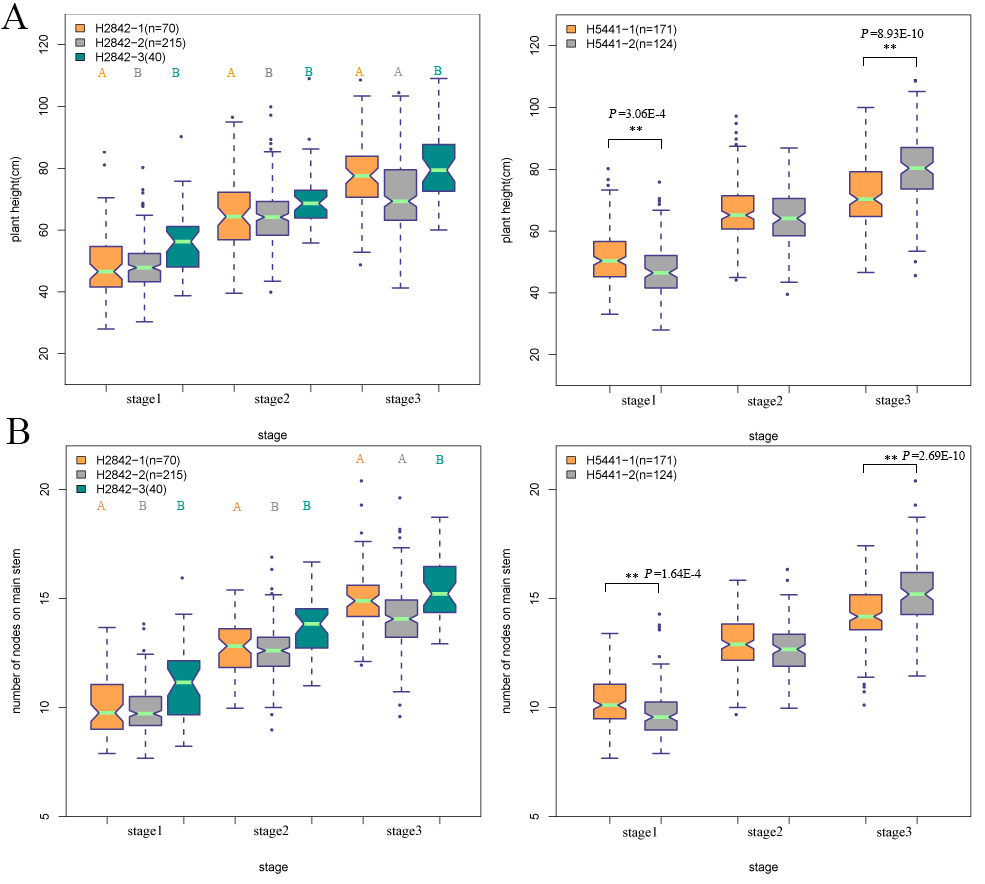
**
